# Effects of Apple and Apple‐Derived Products on Serum Inflammatory Biomarkers, Adiponectin, and Liver Enzymes in Adults: A Systematic Review and Meta‐Analysis of Randomized Controlled Trials

**DOI:** 10.1002/fsn3.72547

**Published:** 2026-11-11

**Authors:** Sahel Hamednia, Mehdi Karimi, Camellia Akhgarjand, Elaheh Dehghani, Melina Todehkharman, Mohammad Mehdi Khaleghi, Omid Asbaghi, Ali Hosseini, Moslem Naderian

**Affiliations:** ^1^ Department of Exercise Physiology, Faculty of Physical Education and Sport Science University of Tabriz Tabriz Iran; ^2^ Faculty of Medicine Bogomolets National Medical University (NMU) Kyiv Ukraine; ^3^ Faculty of Health, Center for Aging Research and Translation University of Canberra Bruce Australian Capital Territory Australia; ^4^ Department of Clinical Nutrition, School of Nutrition and Dietetics Tehran University of Medical Sciences (TUMS) Tehran Iran; ^5^ Faculty of Dentistry Russian University of Medicine Moscow Russia; ^6^ Department of Exercise Physiology, Faculty of Sport Sciences Shahid Chamran University of Ahvaz Ahvaz Iran; ^7^ Cancer Research Center Shahid Beheshti University of Medical Sciences Tehran Iran; ^8^ Department of Pharmacognosy, School of Pharmacy Shiraz University of Medical Sciences Shiraz Iran; ^9^ Medicinal Plants Research Center Yasuj University of Medical Sciences Yasuj Iran

**Keywords:** adiponectin, apple, inflammation, liver enzymes, *Malus domestica*, meta‐analysis, review

## Abstract

Chronic low‐grade inflammation, adipokine dysregulation, and elevated liver enzymes are associated with metabolic disease. Given their rich content of polyphenols, dietary fiber, and other bioactive compounds, apples and apple‐derived products may modulate these biomarkers, although trial findings remain inconsistent. An advanced database search was conducted up to September 1, 2026, to identify eligible randomized controlled trials (RCTs). Outcomes of interest included C‐reactive protein (CRP), interleukin‐6 (IL‐6), tumor necrosis factor‐alpha (TNF‐α), adiponectin, alanine aminotransferase (ALT), aspartate aminotransferase (AST), and alkaline phosphatase (ALP). Pooled effect sizes were calculated as weighted mean differences (WMDs) with 95% confidence intervals (CIs) using a random‐effects model. A total of 19 RCTs were included in the meta‐analysis. The pooled analysis showed that apple and apple‐derived products did not significantly affect CRP (WMD: −0.03 mg/L; *p* = 0.388), IL‐6 (WMD: −0.14 pg/mL; 95% CI: −0.31, 0.02; *p* = 0.082), TNF‐α (WMD: −0.09 pg/mL; *p* = 0.432), adiponectin (WMD: 0.22 mg/dL; *p* = 0.450), ALT (WMD: −0.92 IU/L; *p* = 0.234), ALP (WMD: −2.81 IU/L; *p* = 0.089). However, a significant reduction was observed for AST (WMD: −1.56 IU/L; *p* = 0.006). Exploratory subgroup findings showed that some benefits were observed more frequently in specific contexts, including particular product forms (e.g., extracts or vinegar), longer intervention duration, and specific participant subgroups. Apple and apple‐derived products did not significantly affect most inflammatory biomarkers, adiponectin, or liver enzymes, except for a significant reduction in AST. Given the low to very low certainty of evidence for most outcomes, further well‐designed RCTs are needed to clarify their potential effects.

## Graphical abstract

Summary of the systematic review and meta‐analysis of 19 randomized controlled trials evaluating the effects of apple and apple‐derived products on inflammatory biomarkers, adiponectin, and liver enzymes in adults. Apple interventions significantly reduced AST, whereas no statistically significant effects were observed for CRP, IL‐6, TNF‐α, adiponectin, ALT, or ALP.

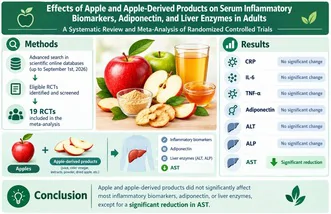

**Abbreviations**
ALP: alkaline phosphatase
ALT: alanine aminotransferase
AST: aspartate aminotransferase
CI: confidence interval
CRP: C‐reactive protein
IL‐6: interleukin‐6
RCT: randomized controlled trial
TNF‐α: tumor necrosis factor‐alpha
WMD: weighted mean difference

## 1 Introduction

Chronic low‐grade inflammation contributes to the development and progression of numerous non‐communicable and metabolic diseases (Cifuentes et al. 2024). Circulating inflammatory biomarkers, such as C‐reactive protein (CRP), interleukin‐6 (IL‐6), and tumor necrosis factor‐alpha (TNF‐α), are established indicators of systemic inflammation and are closely associated with cardiometabolic risk (Ruan et al. 2024). In addition, adipokines (Kirichenko et al. 2022; Clemente‐Suárez et al. 2023) and liver enzymes, including alanine aminotransferase (ALT), aspartate aminotransferase (AST), and alkaline phosphatase (ALP), are important biomarkers of metabolic homeostasis and liver function, with abnormal levels linked to obesity, insulin resistance, and chronic liver disease (Clemente‐Suárez et al. 2023; Liu et al. 2021). Consequently, identifying safe, effective, and readily accessible dietary interventions that can modulate these pathophysiological pathways has become a major focus of clinical nutrition and preventive medicine. Growing evidence indicates that dietary interventions and bioactive food‐derived products can improve inflammatory and cardiometabolic health, highlighting their potential as complementary strategies for preventing and managing metabolic disorders (Setayesh et al. 2025; Karimi, Javadi, Sharifi, et al. 2025; Karimi, Javadi, Hamzavi, et al. 2025; Karimi et al. 2026).

Apple ( *Malus domestica* L.) is one of the most frequently consumed fruits globally, and it is recognized not only for its availability and sensory appeal but also as a significant source of bioactive compounds in the human diet (Garmo et al. 2026; Wang et al. 2014). The health‐promoting effects of apples are mostly attributed to a diverse profile of polyphenols, such as flavan‐3‐ols, hydroxycinnamic acids, dihydrochalcones, flavonols, and anthocyanins, as well as soluble fiber (pectin) (Bars‐Cortina et al. 2020; Lee et al. 2003).

Findings of experimental studies indicate that these apple‐derived polyphenols are believed to enhance bioactivity, exerting pleiotropic effects such as the inhibition of reactive oxygen species (ROS) production, the modulation of cell signaling cascades involved in inflammatory cytokine secretion, and the improvement of endothelial function by mediated pathways of nitric oxide (Bondonno et al. 2018; Del Bo et al. 2015). In addition, specific components such as pectin can enhance the gut microbiota, leading to improved lipid metabolism, glycemic control, and systemic inflammation through complex gut‐brain signaling (Koutsos et al. 2017; Koutsos et al. 2015).

While epidemiological studies consistently report an inverse association between apple intake and the risk of chronic metabolic and cardiovascular diseases (Hodgson et al. 2016; Sandoval‐Ramírez et al. 2020), findings from human clinical trials remain inconsistent (Auclair et al. 2010; Ravn‐Haren et al. 2013). Discrepancies in the literature may stem from heterogeneity in the types of apple products, including whole fruit, skin, and pomace, as well as apple juice and purified extracts, as well as variations in dosage, study duration, and the metabolic populations investigated (Koutsos et al. 2020; Pedret et al. 2024). Although individual trials have explored the impact of apple consumption on specific outcomes, there is a lack of comprehensive evidence synthesizing these effects on the triad of serum inflammatory biomarkers, adiponectin, and liver enzymes (Hollands et al. 2018; Joseph et al. 2016).

A recent systematic review by Del Bo' et al. (2026) reviewed 38 human intervention studies and found that apples and apple‐derived products may improve several cardiometabolic and functional markers, although substantial heterogeneity limited definitive conclusions. However, their review did not quantitatively synthesize evidence specifically for inflammatory biomarkers, adiponectin, and liver enzymes.

Given apples' potential as an affordable, accessible dietary strategy, a comprehensive evaluation of their clinical effects is needed to better inform evidence‐based nutritional recommendations. Therefore, this systematic review and meta‐analysis of randomized controlled trials (RCTs) aimed to assess the effects of apples and/or apple‐derived products on serum inflammatory biomarkers, adiponectin, and liver enzymes in adults. Additionally, this study explored potential sources of variability across studies and provided insights into factors that may influence the effectiveness of these interventions.

## 2 Methods

### 2.1 Study Protocol and Registration

This systematic review and meta‐analysis was conducted in accordance with the *Cochrane Handbook for Systematic Reviews of Interventions* (Higgins et al. 2019) and reported following the Preferred Reporting Items for Systematic Reviews and Meta‐Analyses (PRISMA) guidelines (Page et al. 2021). The review protocol was prospectively registered in the International Prospective Register of Systematic Reviews (PROSPERO Registration No. CRD420261463680). The research question and eligibility criteria were developed using the PICOS (Population, Intervention, Comparison, Outcomes, and Study Design) framework (Amir‐Behghadami and Janati 2020). The PICOS framework and detailed inclusion and exclusion criteria used to determine study eligibility are presented in Table 1.

**TABLE 1:** PICOS framework and inclusion/exclusion criteria.

PICOS domain	Inclusion criteria	Exclusion criteria
Population (P)	Adults aged ≥ 18 years, regardless of sex, geographical region, baseline health status, or comorbidities	Participants aged < 18 years
Intervention (I)	Apple or apple‐derived products, including whole apples, apple juice, apple vinegar, apple extracts, or other apple‐based preparations, administered as the sole intervention	Interventions in which apple products were combined with other active ingredients and their independent effects could not be isolated
Comparison (C)	Placebo, control group, no supplementation, or no‐intervention group	Uncontrolled or non‐randomized intervention studies
Outcomes (O)	At least one predefined outcome: CRP, IL‐6, adiponectin, ALT, AST, or ALP	Studies reporting none of the predefined outcomes
Study design (S)	RCT with parallel‐group or crossover designs	Observational studies (cohort, case–control, cross‐sectional, or ecological), case reports, reviews, editorials, letters, conference abstracts, non‐randomized studies, and uncontrolled intervention studies
Quantitative data	Sufficient numerical data to calculate effect sizes	Insufficient data for quantitative synthesis after attempts to obtain additional information
Publication and study availability	Full‐text studies meeting all other eligibility criteria; no restrictions on publication year or language	Duplicate publications (only the most complete report retained), inaccessible full texts, and gray literature (e.g., dissertations, theses, conference proceedings, and unpublished studies), which was not searched

Abbreviations: ALP, alkaline phosphatase; ALT, alanine aminotransferase; AST, aspartate aminotransferase; CRP, C‐reactive protein; IL‐6, interleukin‐6; RCT, randomized controlled trial.

### 2.2 Eligibility Criteria

Studies were considered eligible if they met all of the following inclusion criteria: (1) RCTs (parallel or crossover design); (2) conducted in adults aged ≥ 18 years; (3) evaluated the effects of apple or apple‐derived products (e.g., whole apples, apple juice, apple vinegar, apple extracts, or other apple‐based preparations) administered as the sole intervention; (4) included a placebo, control, or no‐treatment group for comparison; (5) reported data for at least one of the predefined outcomes; and (6) provided sufficient quantitative data to calculate effect sizes. No restrictions were imposed regarding publication year, language, sex, geographical region, baseline health status, or comorbidities.

Studies were excluded if they met any of the following criteria: (1) observational studies (cohort, case–control, cross‐sectional, or ecological studies), case reports, conference abstracts, review articles, editorials, letters, or animal and in vitro studies; (2) non‐randomized or uncontrolled intervention studies; (3) studies involving participants < 18 years of age; (4) interventions in which the independent effect of apple products could not be isolated because they were combined with other active ingredients; (5) studies that did not report any of the predefined outcomes or lacked sufficient data for quantitative synthesis after attempts to obtain additional information; (6) duplicate publications, for which only the most complete report was retained. Gray literature (e.g., dissertations, theses, conference proceedings, and unpublished studies) was not searched, and studies with inaccessible full texts were therefore not considered for inclusion.

### 2.3 Search Strategy

A comprehensive and systematic literature search was conducted to identify all relevant RCTs. Electronic databases, including PubMed/MEDLINE, Scopus, and Web of Science, were searched from the inception of each database through September 1st 2026, without restrictions on publication year or language. The search strategy incorporated keywords and synonyms related to apple, inflammatory biomarkers, adiponectin, liver enzymes, and RCT. Boolean operators (AND/OR), truncation symbols, and database‐specific search filters were applied as appropriate to maximize sensitivity. The detailed search keywords and search strategies for all three databases are provided separately in Supplementary Tables 1 and 2. To ensure comprehensive study identification, *Google Scholar* and the reference lists of all eligible articles and relevant review papers were manually screened for additional studies that may have been missed during the electronic database search.

### 2.4 Data Extraction

Two reviewers (S.H. and M.M.K.) independently extracted data from all eligible studies using a standardized data extraction form. Any discrepancies between reviewers were resolved through discussion and, when necessary, consultation with a third reviewer (O.A.) until consensus was reached. The following information was extracted from each included trial: first author, publication year, country, study design, sample size, participant characteristics (including mean age, sex distribution, body mass index [BMI], and health status), details of the intervention (type of apple or apple‐derived product, dosage, and duration), characteristics of the control group, and outcome data. For each outcome of interest, baseline and post‐intervention means, mean changes (when available), corresponding measures of variability (standard deviation [SD], standard error [SE], or 95% confidence interval [CI]), and the number of participants analyzed were extracted for serum inflammatory biomarkers (CRP, IL‐6, and TNF‐α), adiponectin, and liver enzymes (ALT, AST, and ALP). When outcome data were incomplete or reported in an unsuitable format, appropriate statistical conversions were performed according to the *Cochrane Handbook for Systematic Reviews of Interventions* (Higgins et al. 2019) to obtain comparable effect estimates for the meta‐analysis.

### 2.5 Statistical Analysis

All statistical analyses were performed using Stata software, version 19 (Stata Corp., College Station, TX), with statistical significance defined as a two‐tailed *p*‐value < 0.05. To synthesize the findings, a random‐effects model was employed to calculate pooled weighted mean differences (WMDs) with corresponding 95% confidence intervals (CIs), thereby accounting for potential between‐study heterogeneity (DerSimonian and Laird 1986). Mean difference outcomes were determined by comparing changes from baseline to post‐intervention between the intervention and comparison groups. For studies omitting the standard deviation (SD) of the mean change, SDs were calculated using the following Cochrane‐approved formula (Borenstein et al. 2009):

$$SD_{\text{change}}=\sqrt{SD_{\text{baseline}}^{2}+SD_{\text{final}}^{2}-\left(2\times r\times SD_{\text{baseline}}\times SD_{\text{final}}\right)}$$

When the SD of the change score was unavailable, it was calculated using a standard formula and a correlation coefficient (*r*) of 0.8. SEs, 95% CIs, and IQRs were converted to SDs using the approach described by Hozo et al. (Hozo et al. 2005). Meta‐analyses used a random‐effects model to account for between‐study heterogeneity. Heterogeneity was assessed with Cochran's *Q* test and quantified using the *I* ^2^ statistic. In accordance with Cochrane guidance (Ioannidis 2008), *I* ^2^ values of 0%–40%, 40%–60%, 60%–80%, and 80%–100% were interpreted as indicating low, moderate, substantial, and considerable heterogeneity, respectively (Ioannidis 2008).

For crossover RCTs, data from the intervention and comparison periods were treated as paired observations when sufficient within‐participant variance information was available; where paired variance estimates were not reported, the available variance data were used according to the information provided in the original study.

To explore potential sources of heterogeneity, pre‐specified exploratory subgroup analyses were conducted based on participant characteristics including geographical region, sex, baseline BMI categories (kg/m^2^), trial duration (< 8 weeks vs. ≥ 8 weeks), and intervention type (apple extract vs. apple vinegar).

Furthermore, random‐effects meta‐regression and non‐linear dose–response regression analyses were conducted to evaluate potential moderating effects of intervention study duration on the specified outcomes, thereby facilitating the synthesis of correlated dose–response data across studies (Xu and Doi 2018; Xie et al. 2021). Potential publication bias was rigorously assessed using visual inspection of funnel plots, Egger's linear regression, and Begg's rank correlation tests, with the trim‐and‐fill method applied to address any detected asymmetry (Shi and Lin 2019). Finally, the robustness of the pooled estimates was evaluated through sensitivity analyses using the leave‐one‐out method (Begg and Mazumdar 1994; Egger et al. 1997).

### 2.6 Risk of Bias Assessment

Risk of bias in the included papers was independently evaluated by two authors (S.H. and M.M.K.) using the RoB‐2 tool (the revised Cochrane risk‐of‐bias tool for randomized trials). RoB‐2 assesses bias in five distinct domains: the randomization process, deviations from intended interventions, missing outcome data, measurement of the outcome, and selection of the reported result. The judgments within each domain lead to a general risk of bias: “low risk of bias,” “some concerns,” or “high risk of bias” (Sterne et al. 2019).

### 2.7 Certainty of Evidence Assessment

The overall certainty of evidence across RCTs was evaluated separately by two reviewers (O.A. and M.K.) utilizing the GRADE guidelines (Guyatt et al. 2008). As a result, the quality of evidence was classified into four categories: high, moderate, low, and very low. These criteria ensured a standardized and transparent assessment of the strength of the evidence.

## 3 Results

### 3.1 Study Selection

PRISMA Flow chart of the study selection process for included studies in the systematic review is illustrated in Figure 1. A total of 770 records were identified through database searching, including PubMed (*n* = 125), Web of Science (*n* = 257), and Scopus (*n* = 388). After removing 191 duplicate records, 579 articles remained for title and abstract screening. Of these, 555 records were excluded because they were review articles, non‐human studies, or had irrelevant titles or abstracts. The full texts of 24 potentially eligible studies were assessed, and five were subsequently excluded due to non‐randomized design, insufficient outcome data, or lack of an appropriate control group. Ultimately, 19 studies (Auclair et al. 2010; Ravn‐Haren et al. 2013; Koutsos et al. 2020; Pedret et al. 2024; Akazome et al. 2010; Alzoufairi et al. 2025; Barnett et al. 2021; Barth et al. 2012; Chai et al. 2012; Cicero et al. 2017; Gasper et al. 2014; Gheflati et al. 2019; Guo et al. 2008; Liddle et al. 2021; Nagasako‐Akazome et al. 2007; Panetta et al. 2013; Pushpass et al. 2023; Saarenhovi et al. 2017; Tenore et al. 2018) met the eligibility criteria and were included in the systematic review and meta‐analysis.

**FIGURE 1:**
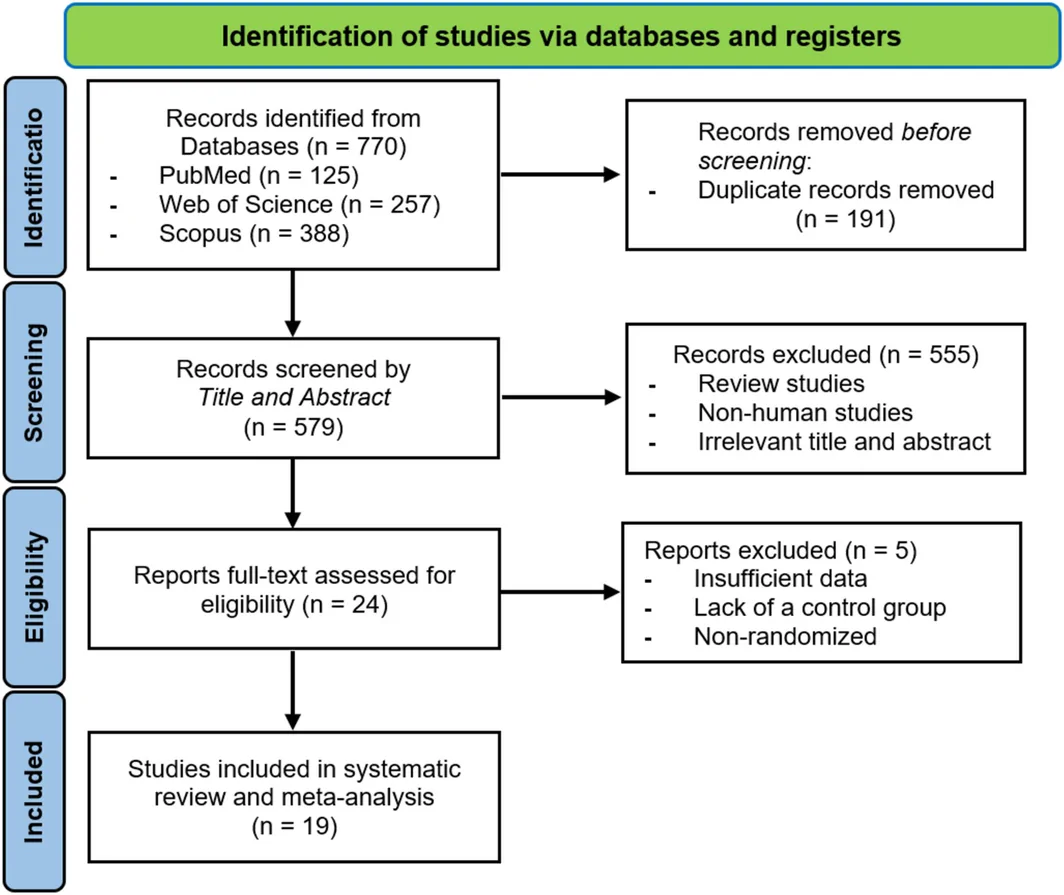
PRISMA Flow chart of the study selection process for the inclusion of randomized controlled trials (RCTs) in the systematic review and meta‐analysis of the effects of apples and apple‐derived products on serum inflammatory biomarkers, adiponectin, and liver enzymes in adults.

### 3.2 Study Characteristics

A total of 19 RCTs (Auclair et al. 2010; Ravn‐Haren et al. 2013; Koutsos et al. 2020; Pedret et al. 2024; Akazome et al. 2010; Alzoufairi et al. 2025; Barnett et al. 2021; Barth et al. 2012; Chai et al. 2012; Cicero et al. 2017; Gasper et al. 2014; Gheflati et al. 2019; Guo et al. 2008; Liddle et al. 2021; Nagasako‐Akazome et al. 2007; Panetta et al. 2013; Pushpass et al. 2023; Saarenhovi et al. 2017; Tenore et al. 2018) comprising 24 intervention arms were included in this systematic review (Table 2). The studies were published between 2007 and 2025 and were conducted across diverse geographical regions, including Japan, China, France, Germany, Denmark, the USA, the UK, Finland, Italy, Iran, Canada, New Zealand, Ireland, and Spain. Most trials employed a parallel‐group design, while the remainder used a crossover design, with the majority being placebo‐controlled and several incorporating double‐ or single‐blind methodologies. The studies enrolled a broad range of participants, including healthy adults, overweight or obese individuals, patients with hypercholesterolemia, type 2 diabetes mellitus with dyslipidemia, borderline or mild hypertension, and non‐diabetic obese participants. Sample sizes ranged from 29 to 100 participants, with intervention groups ranging from 13 to 57 participants, and the mean age of participants ranged from approximately 36 to 64 years. Baseline BMI ranged from 20.8 to 33.3 kg/m^2^, representing normal‐weight, overweight, and obese populations. Intervention durations ranged from 10 days to 52 weeks, although most studies lasted between 4 and 12 weeks. A wide variety of apple‐derived interventions were evaluated, including whole apples, dried apples, lyophilized apples, apple juice (clear and cloudy), apple puree, apple pomace, apple cider vinegar/apple vinegar, and apple polyphenol extracts (including Annurca apple and *Malus domestica* extracts), with daily doses varying according to the intervention type. Control groups received placebo preparations, no treatment, alternative apple varieties, sugar‐matched or isocaloric control beverages, dried plums, pomegranate juice, aspirin, or cornflakes with milk plus placebo capsules, depending on the study design.

**TABLE 2:** Characteristics of the 19 included randomized controlled trials (RCTs) in the systematic review and meta‐analysis of the effects of apples and apple‐derived products on serum inflammatory biomarkers, adiponectin, and liver enzymes in adults.

Studies	Country	Study design	Health status of participant	Sex	Sample size		Trial duration (week)	Age (year)		BMI (kg/m^2^)		Intervention	
					IG	CG		IG	CG	IG	CG	IG	CG
Nagasako‐Akazome et al. (2007)	Japan	RCT, Parallel, DB, PC	Healthy and moderately obese	M, F	24	24	12	47.8 ± 10.8	46.5 ± 10.8	25.9 ± 1.9	25.9 ± 2.2	Apple polyphenol extract (600 mg/day)	Placebo
Guo et al. (2008)	China	RCT, Parallel	Elderly	M: 20, F: 6	13	13	4	62.8 ± 4.7	64.1 ± 4.3	24.2 ± 2.5	23.0 ± 2.5	Apple juice (250 mL/day)	Pomegranate
Auclair et al. (2010)	France	RCT, Crossover, DB	Hyper‐cholesterolemia	M: 30	30	30	4	52.6 ± 5.5	52.6 ± 5.5	25.7 ± 2.6	25.7 ± 2.6	Lyophilized apples (Marie Me'nard apple) (40 g/day)	Golden Delicious apple
Akazome et al. (2010)	Japan	RCT, Parallel, DB, PC	Healthy	M: 56, F: 38	47	47	12	45.3 ± 8.6	44.9 ± 9.1	27.1 ± 1.5	27.0 ± 1.4	Apple polyphenol extract (340 g/bottle (containing 600 mg apple polyphenols) three times/day)	Placebo
Akazome et al. (2010)	Japan	RCT, Parallel, DB, PC	Healthy	M: 20, F: 10	15	15	4	40.4 ± 10.4	41.6 ± 9.8	23.3 ± 3.0	23.4 ± 2.9	Apple polyphenol extract (340 g/bottle (containing 600 mg apple polyphenols) three times/day)	Placebo
Chai et al. (2012)	USA	RCT, Parallel	Healthy postmenopausal women	F:160	45	55	52	55.6 ± 5.0	57.5 ± 4.01	24.8 ± 4.1	24.9 ± 4.6	Dried apple (75 g/day)	100 g dried plum
Barth et al. (2012)	Germany	RCT, Parallel, SB	Non‐diabetic obese men	M: 68	38	30	4	49 (23–69)	49 (23–69)	31.1 ± 3.6	30.5 ± 3.0	Cloudy apple juice (750 mL/day)	750 mL of control beverage (isocaloric)/day
Ravn‐Haren et al. (2013)	Denmark	RCT, Crossover, SB	Healthy	M: 9, F: 14	23	23	20	36.2 ± 17.9	36.2 ± 17.9	22.3 ± 2.59	22.3 ± 2.59	Whole fresh apples (550 g/day)	No treatment
Ravn‐Haren et al. (2013)	Denmark	RCT, Crossover, SB	Healthy	M: 9, F: 14	23	23	20	36.2 ± 17.9	36.2 ± 17.9	22.3 ± 2.59	22.3 ± 2.59	Apple pomace (22 mg/day)	No treatment
Ravn‐Haren et al. (2013)	Denmark	RCT, Crossover, SB	Healthy	M: 9, F: 14	23	23	20	36.2 ± 17.9	36.2 ± 17.9	22.3 ± 2.59	22.3 ± 2.59	Cloudy apple juice (500 mL/day)	No treatment
Ravn‐Haren et al. (2013)	Denmark	RCT, Crossover, SB	Healthy	M: 9, F: 14	23	23	20	36.2 ± 17.9	36.2 ± 17.9	22.3 ± 2.59	22.3 ± 2.59	Clear apple juice (500 mL/day)	No treatment
Panetta et al. (2013)	USA	RCT, Parallel, DB, PC	Non‐diabetics	M: 63, F: 34	50	47	8	57.7 ± 9.33	56.1 ± 12.58	28.3 ± 5.87	26.8 ± 4.19	Apple cider vinegar (30 mL/day)	Placebo
Gasper et al. (2014)	UK	RCT, Crossover	Healthy	M: 13, F: 12	25	25	4	42 ± 11	42 ± 11	25.9 ± 4.2	25.9 ± 4.2	Apple puree (230 g/day [containing 100 mg epicatechin])	Aspirin (75 mg/day)
Gasper et al. (2014)	UK	RCT, Crossover	Healthy	M: 13, F: 12	26	25	4	42 ± 11	42 ± 11	25.9 ± 4.2	25.9 ± 4.2	Apple puree (230 g/day [containing 25 mg epicatechin])	Aspirin (75 mg/day)
Saarenhovi et al. (2017)	Finland	RCT, Crossover, DB, PC	Borderline hypertension or unmedicated mild hypertension	M: 26, F: 34	57	57	4	55.6 ± 7.9	54.9 ± 6.4	24.7 ± 3.0	26.3 ± 3.5	Apple polyphenol extract (330 mg/day (containing 100 mg of epicatechin))	Placebo
Cicero et al. (2017)	Italy	RCT, parallel, DB, PC	Overweight (BMI > 25)	M, F	31	31	8	63.3 ± 7.5	63.2 ± 11.6	25–30	25–30	Apple polyphenol extract ( *Malus Domestica* ) (300 mg/day)	Placebo
Tenore et al. (2018)	Italy	RCT, Crossover, TB, PC	Healthy	M: 30, F: 20	50	50	10 days	43.5 ± 10.2	43.5 ± 10.2	20.8 ± 3.1	20.8 ± 3.1	Annurca apple polyphenolic extract (1000 mg/day)	Placebo
Gheflati et al. (2019)	Iran	Parallel, C	T2DM & dyslipidemia	M: 20, F: 42	32	30	8	49.47 ± 8.02	52.1 ± 7.87	28.96 ± 4.31	28.85 ± 4.24	Apple vinegar (20 mL/day)	No treatment
Koutsos et al. (2020)	UK	RCT, Crossover	Healthy with mild hyper‐cholesterolemia	M: 17, F: 23	40	40	8	51 ± 11	51 ± 11	25.3 ± 3.7	25.3 ± 3.7	Whole apple (2 apples [340 g]/day)	500 mL of sugar‐matched apple control beverage
Liddle et al. (2021)	Canada	RCT, Parallel, PC	Obese and overweight	M: 14, F: 30	22	22	6	48 ± 14.54	42.9 ± 15.0	33.2 ± 6.09	33.3 ± 5.62	Whole Gala apple (600 g [3 whole Gala apples (∼200 g edible parts)/day])	Placebo
Barnett et al. (2021)	New Zealand	RCT, Crossover	Healthy	M: 5, F: 20	25	25	2	F: 39.8, M: 47.3	F: 38, M: 39.7	F: 23.3, M: 22.8	F: 23.4, M: 27.4	Red‐fleshed apple (1 portion (equivalent to 1 apple) daily)	White‐fleshed apple
Pushpass et al. (2023)	Ireland	RCT, Parallel, DB, C	Healthy	M: 15, F: 46	16	15	8	55 ± 16	49 ± 15.49	25.7 ± 3.87	24.9 ± 3.65	Renetta apple (2 apples/day)	40 g Cornflakes with 180 mL semi‐skimmed milk and 2 placebo capsules
Pedret et al. (2024)	Spain	RCT, Parallel, C	Hyper‐cholesterolemia	M: 51, F: 70	40	41	6	46.7 ± 16.3	49.8 ± 13.6	26.3 ± 3.8	24.6 ± 3.2	Red‐fleshed apples (80 g/day)	White‐fleshed apples
Alzoufairi et al. (2025)	UK	RCT, Parallel, PC	Healthy	M: 14, F: 45	15	14	8	54 ± 3	50 ± 3	25.5 ± 0.9	24.7 ± 0.9	Renetta Canada apples (2 Renetta Canada apples/day with 2 placebo capsules)	Placebo

Abbreviations: BMI, body mass index; CG, control group; DB, double blind; F, female; IG, intervention group; M, male; NR, not reported; PC, placebo‐controlled; RCT, randomized controlled trial; T2DM, type 2 diabetes mellitus.

### 3.3 Risk of Bias Assessment

The risk of bias assessment of the 19 included RCTs (Auclair et al. 2010; Ravn‐Haren et al. 2013; Koutsos et al. 2020; Pedret et al. 2024; Akazome et al. 2010; Alzoufairi et al. 2025; Barnett et al. 2021; Barth et al. 2012; Chai et al. 2012; Cicero et al. 2017; Gasper et al. 2014; Gheflati et al. 2019; Guo et al. 2008; Liddle et al. 2021; Nagasako‐Akazome et al. 2007; Panetta et al. 2013; Pushpass et al. 2023; Saarenhovi et al. 2017; Tenore et al. 2018) is presented in Table 3. Overall, 10 RCTs were judged to have a high risk of bias, 8 RCTs were rated as having some concerns, and only 1 RCT was considered to have a low risk of bias. The most frequent sources of bias were related to the randomization process (Domain 1), deviations from intended interventions (Domain 2), and missing outcome data (Domain 3). In contrast, the majority of studies were judged to be at low risk of bias for the measurement of outcomes (Domain 4) and the selection of the reported results (Domain 5). Overall, these findings indicate that methodological limitations were common among the included trials and should be considered when interpreting the pooled estimates.

**TABLE 3:** Risk of bias (RoB) assessment of the included 19 randomized controlled trials (RCTs) in the systematic review and meta‐analysis of the effects of apples and apple‐derived products on serum inflammatory biomarkers, adiponectin, and liver enzymes in adults.

Studies	D1	D2	D3	D4	D5	Overall RoB
Nagasako‐Akazome et al. (2007)	High	Low	Low	Low	High	High
Guo et al. (2008)	High	High	Low	Low	Low	High
Auclair et al. (2010)	High	Low	Low	Low	Low	Some concerns
Akazome et al. (2010)	High	Low	Low	Low	Low	Some concerns
Chai et al. (2012)	Low	High	High	Low	Low	High
Barth et al. (2012)	High	Low	High	Low	Low	High
Ravn‐Haren et al. (2013)	High	High	Low	Low	Low	High
Panetta et al. (2013)	Low	Low	High	Low	Low	Some concerns
Gasper et al. (2014)	High	High	Low	Low	Low	High
Saarenhovi et al. (2017)	High	Low	High	Low	Low	High
Cicero et al. (2017)	Low	Low	High	Low	Low	Some concerns
Tenore et al. (2018)	Low	Low	Low	Low	Low	Low
Gheflati et al. (2019)	Low	High	High	Low	Low	High
Koutsos et al. (2020)	Low	High	Low	Low	Low	Some concerns
Liddle et al. (2021)	Low	High	Low	Low	Low	Some concerns
Barnett et al. (2021)	High	High	High	Low	Low	High
Pushpass et al. (2023)	Low	Low	High	Low	Low	Some concerns
Pedret et al. (2024)	Low	High	High	Low	Low	High
Alzoufairi et al. (2025)	High	High	High	Low	Low	High

*Note:* D1, bias arising from the randomization process; D2, bias due to deviations from the intended interventions; D3, bias due to missing outcome data; D4, bias in measurement of the outcome; D5, bias in selection of the reported result. Overall risk of bias was classified as **low risk** when no domains were judged to have a high risk of bias, **some concerns** when one domain was judged to have a high risk of bias, and **high risk** when more than one domain was judged to have a high risk of bias.

### 3.4 Meta‐Analysis

#### 3.4.1 Effect on C‐Reactive Protein

A pooled analysis of 16 intervention arms from 13 RCTs demonstrated that apple product consumption did not significantly affect circulating CRP concentrations compared with the control groups (WMD = −0.03 mg/L, 95% CI: −0.08 to 0.03, *p* = 0.388). Moderate between‐study heterogeneity was observed (*I* ^2^ = 59.1%) (Figure 2A).

**FIGURE 2:**
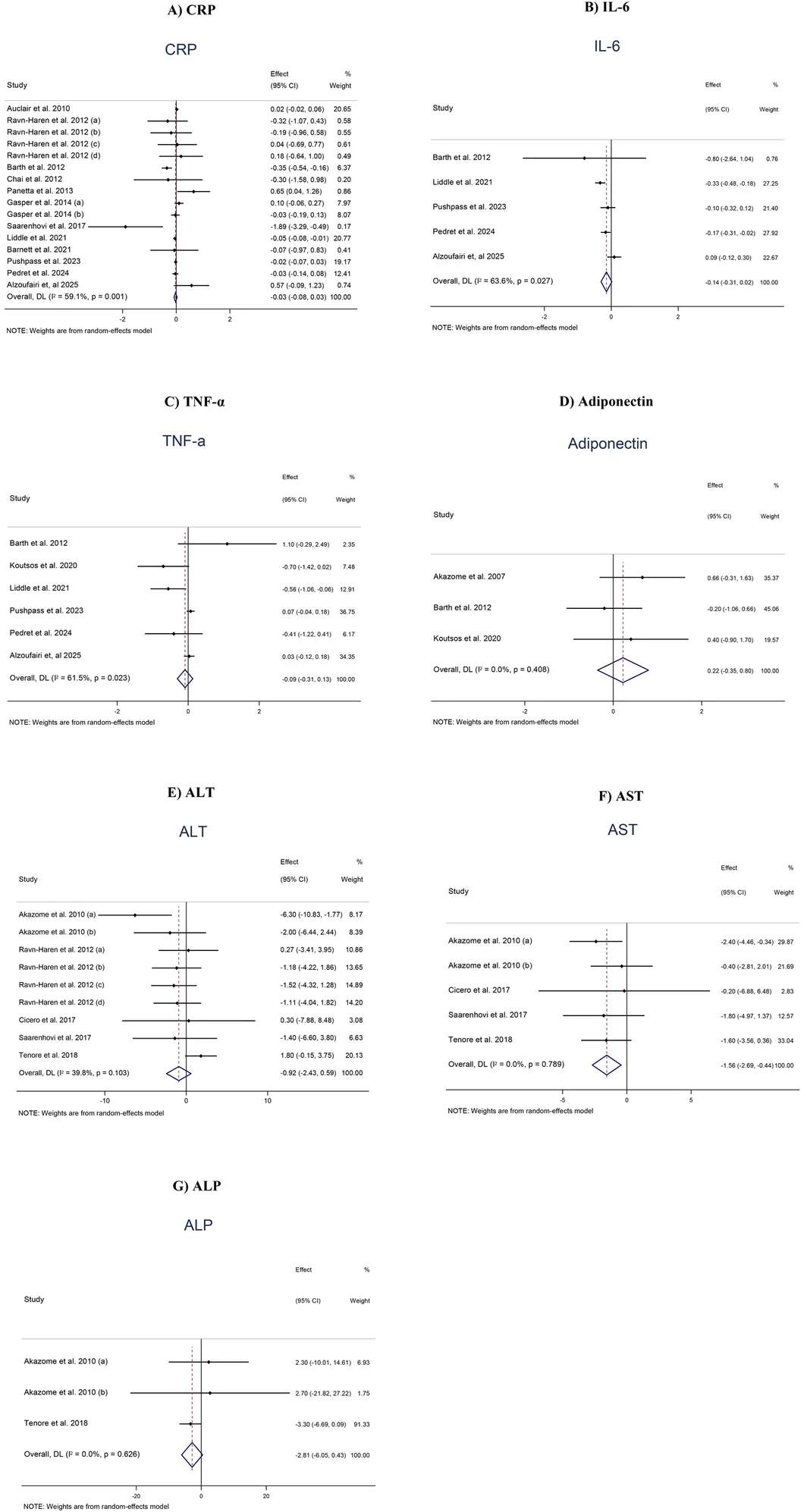
Forest plot detailing weighted mean difference (WMD) and 95% confidence intervals (CIs) for the effect of apple products on: (A) CRP (mg/dL), (B) IL‐6 (pg/mL), (C) TNF‐α (pg/mL), (D) Adiponectin (mg/dL), (E) ALT (IU/L), (F) AST (IU/L), and (G) ALP (IU/L).

Exploratory subgroup analyses revealed no significant effect according to baseline CRP concentration, geographical region, sex, intervention duration, or baseline BMI. However, significant effects were observed in studies evaluating apple extract (WMD = −1.89 mg/L, *p* = 0.008) and apple vinegar (WMD = 0.65 mg/L, *p* = 0.037), although these findings should be interpreted cautiously because they were derived from a single study (Table 4).

**TABLE 4:** Overall and subgroup meta‐analysis findings for the effect of apple products on serum biomarkers of inflammation, adiponectin and liver enzymes in adults.

	Number of ES	WMD (95% CI)	*p*	Heterogeneity	
				*p* heterogeneity	*I* ^2^
*Subgroup analyses of Apple products on CRP (mg/L)*					
Overall effect	16	−0.03 (−0.08, 0.03)	0.388	0.001	59.1%
Baseline CRP (mg/L)					
> 3	2	0.03 (−0.09, 0.16)	0.586	0.256	22.5%
< 3	5	−0.04 (−0.10, 0.02)	0.182	0.001	78.7%
Geographical region					
America	3	0.14 (−0.38, 0.66)	0.598	0.075	61.4%
Europe	13	−0.02 (−0.10, 0.04)	0.437	0.004	59.8%
Other	1	−0.07 (−0.96, 0.82)	0.878	—	—
Sex					
Both sexes	13	−0.01 (−0.07, 0.04)	0.657	0.075	38.8%
Female	1	−0.30 (−1.58, 0.98)	0.646	—	—
Male	2	−0.15 (−0.51, 0.21)	0.415	< 0.001	92.7%
Trial duration (week)					
< 8	8	−0.04 (−0.11, 0.03)	0.264	< 0.001	74.8%
≥ 8	8	0.07 (−0.13, 0.27)	0.498	0.265	20.8%
Intervention type					
Apple Extract	1	−1.89 (−3.28, −0.49)	0.008*	—	—
Apple Fruit	9	−0.01 (−0.05, 0.02)	0.378	0.179	30.0%
Apple Juice	3	−0.24 (−0.52, 0.04)	0.093	0.299	17.1%
Apple Puree	2	0.03 (−0.09, 0.16)	0.586	0.256	22.5%
Apple Vinegar	1	0.65 (0.04, 1.26)	0.037*	—	—
Baseline BMI (kg/m^2^)					
Normal (18.5–24.9)	7	−0.20 (−0.57, 0.15)	0.262	0.315	15.1%
Overweight (25–29.9)	7	0.01 (−0.04, 0.06)	0.653	0.103	43.2%
Obese (> 30)	2	−0.18 (−0.47, 0.11)	0.219	0.003	88.9%
*Subgroup analyses of Apple products on IL‐6 (pg/mL)*					
Overall effect	5	−0.14 (−0.31, 0.02)	0.082	0.027	63.6%
Geographical region					
America	1	−0.33 (−0.48, −0.17)	< 0.001*	—	—
Europe	4	−0.07 (−0.22, 0.06)	0.298	0.207	34.2%
Sex					
Both sexes	4	−0.13 (−0.30, 0.02)	0.106	0.014	71.5%
Male	1	−0.80 (−2.64, 1.04)	0.394	—	—
Trial duration (week)					
< 8	3	−0.24 (−0.38, −0.11)	< 0.001*	0.273	22.9%
≥ 8	2	−0.00 (−0.18, 0.18)	0.998	0.223	32.8%
Intervention type					
Apple Fruit	4	−0.13 (−0.30, 0.02)	0.106	0.014	71.5%
Apple Juice	1	−0.80 (−2.64, 1.04)	0.394	—	—
Baseline BMI (kg/m^2^)					
Overweight (25–29.9)	3	−0.07 (−0.22, 0.08)	0.369	0.136	49.8%
Obese (> 30)	2	−0.33 (−0.48, −0.18)	< 0.001*	0.618	0.0%
*Subgroup analyses of Apple products on TNF‐α (pg/mL)*					
Overall effect	6	−0.09 (−0.31, 0.13)	0.432	0.023	61.5%
Geographical region					
America	1	−0.56 (−1.06, −0.05)	0.030*	—	—
Europe	5	0.00 (−0.17, 0.18)	0.963	0.104	47.9%
Sex					
Both sexes	5	−0.10 (−0.31, 0.10)	0.332	0.030	62.5%
Male	1	1.10 (−028, 2.48)	0.120	—	—
Trial duration (week)					
< 8	3	−0.18 (−0.93, 0.56)	0.632	0.088	58.8%
≥ 8	3	0.01 (−0.14, 0.17)	0.847	0.117	53.3%
Intervention					
Apple Fruit	5	−0.10 (−0.31, 0.10)	0.332	0.030	62.5%
Apple Juice	1	1.10 (−028, 2.48)	0.120	—	—
Baseline BMI (kg/m^2^)					
Overweight (25–29.9)	4	−0.00 (−0.16, 0.16)	0.998	0.142	44.9%
Obese (> 30)	2	0.13 (−1.46, 1.74)	0.865	0.028	79.4%
*Subgroup analyses of Apple products on Adiponectin (mg/dL)*					
Overall effect	3	0.22 (−0.35, 0.80)	0.450	0.408	0.0%
Geographical region					
Asia	1	0.66 (−0.30, 1.62)	0.181	—	—
Europe	2	−0.01 (−0.73, 0.69)	0.960	0.450	0.0%
Sex					
Both sexes	2	0.56 (−0.20, 1.34)	0.152	0.753	0.0%
Male	1	−0.20 (−1.05, 0.65)	0.647	—	—
Trial duration (week)					
< 8	1	−0.20 (−1.05, 0.65)	0.647	—	—
≥ 8	2	0.56 (−0.20, 1.34)	0.152	0.753	0.0%
Baseline BMI (kg/m^2^)					
Overweight (25–29.9)	2	0.56 (−0.20, 1.34)	0.152	0.753	0.0%
Obese (> 30)	1	−0.20 (−1.05, 0.65)	0.647	—	—
*Subgroup analyses of Apple products on ALT (IU/L)*					
Overall effect	9	−0.92 (−2.43, 0.59)	0.234	0.103	39.8%
Geographical region					
Asia	2	−4.12 (−8.34, 0.08)	0.055	0.184	43.4%
Europe	7	−0.00 (−1.17, 1.15)	0.988	0.441	0.0%
Trial duration (week)					
< 8	3	0.24 (−2.37, 2.87)	0.852	0.201	37.6%
≥ 8	6	−1.51 (−3.03, 0.00)	0.050	0.357	9.2%
Intervention type					
Apple Extract	5	−1.39 (−4.71, 1.93)	0.411	0.019	66.0%
Apple Fruit	2	−0.59 (−2.93, 1.74)	0.620	0.551	0.0%
Apple Juice	2	−1.32 (−3.34, 0.69)	0.199	0.843	0.0%
Baseline BMI (kg/m^2^)					
Normal (18.5–24.9)	7	−0.22 (−1.43, 0.98)	0.716	0.364	8.5%
Overweight (25–29.9)	2	−3.91 (−10.13, 2.29)	0.217	0.167	47.8%
*Subgroup analyses of Apple products on AST (IU/L)*					
Overall effect	5	−1.56 (−2.69, −0.44)	0.006*	0.789	0.0%
Geographical region					
Asia	2	−1.50 (−3.45, 0.44)	0.131	0.216	34.6%
Europe	3	−1.57 (−3.18, 0.04)	0.057	0.913	0.0%
Trial duration (week)					
< 8	3	−1.25 (−2.62, 0.11)	0.074	0.699	0.0%
≥ 8	2	−2.20 (−4.17, −0.24)	0.028*	0.537	0.0%
Baseline BMI (kg/m^2^)					
Normal (18.5–24.9)	3	−1.25 (−2.62, 0.11)	0.074	0.699	0.0%
Overweight (25–29.9)	2	−2.20 (−4.17, −0.24)	0.028*	0.537	0.0%
*Subgroup analyses of Apple products on ALP (IU/L)*					
Overall effect	3	−2.81 (−6.05, 0.43)	0.089	0.626	0.0%
Geographical region					
Asia	2	2.38 (−8.62, 13.38)	0.671	0.977	0.0%
Europe	1	−3.30 (−6.69, 0.09)	0.056	—	—
Trial duration (week)					
< 8	2	−3.18 (−6.54, 0.17)	0.063	0.635	0.0%
≥ 8	1	2.30 (−10.00, 14.60)	0.714	—	—
Baseline BMI (kg/m^2^)					
Normal (18.5–24.9)	2	−3.18 (−6.54, 0.17)	0.063	0.635	0.0%
Overweight (25–29.9)	1	2.30 (−10.00, 14.60)	0.714	—	—

*Note:* Bold values with an asterisk (*) indicate statistically significant (*p* < 0.05).

Abbreviations: ALP, alkaline phosphatase; ALT, alanine aminotransferase; AST, aspartate aminotransferase; CI, confidence interval; CRP, C‐reactive protein; ES, effect size; IL‐6, interleukin‐6; TNF‐α, tumor necrosis factor‐alpha; WMD, weighted mean difference.

#### 3.4.2 Effect on Interleukin‐6

Five RCTs were included in the pooled analysis of IL‐6. Apple product supplementation did not significantly reduce circulating IL‐6 levels compared with controls (WMD = −0.14 pg/mL, 95% CI: −0.31 to 0.02, *p* = 0.082). Moderate heterogeneity was observed among studies (*I* ^2^ = 63.6%) (Figure 2B).

Exploratory subgroup analyses demonstrated significant reductions in studies conducted in American populations (WMD = −0.33 pg/mL, 95% CI: −0.48 to −0.17, *p* < 0.001), in interventions lasting < 8 weeks (WMD = −0.24 pg/mL, 95% CI: −0.38 to −0.11, *p* < 0.001), and among participants with obesity (BMI > 30 kg/m^2^) (WMD = −0.33 pg/mL, 95% CI: −0.48 to −0.18, *p* < 0.001). No significant effects were detected in the remaining subgroup analyses (Table 4).

#### 3.4.3 Effect on Tumor Necrosis Factor‐Alpha

The pooled analysis of six RCTs showed that apple product consumption had no significant effect on circulating TNF‐α concentrations (WMD = −0.09 pg/mL, 95% CI: −0.31 to 0.13, *p* = 0.432), with moderate heterogeneity (*I* ^2^ = 61.5%) (Figure 2C). In exploratory subgroup analyses, a significant reduction was observed only in studies conducted in American populations (WMD = −0.56 pg/mL, 95% CI: −1.06 to −0.05, *p* = 0.030), whereas no significant effects were found according to sex, intervention duration, intervention type, or baseline BMI (Table 4).

#### 3.4.4 Effect on Adiponectin

Three RCTs were available for adiponectin. Overall, apple products did not significantly affect circulating adiponectin concentrations (WMD = 0.22 mg/dL, 95% CI: −0.35 to 0.80, *p* = 0.450). No evidence of between‐study heterogeneity was observed (*I* ^2^ = 0.0%) (Figure 2D). Likewise, subgroup analyses according to geographical region, sex, intervention duration, and baseline BMI showed no statistically significant differences (Table 4).

#### 3.4.5 Effect on Alanine Aminotransferase

Nine intervention arms from five RCTs were included in the meta‐analysis of ALT. Apple product supplementation did not significantly reduce serum ALT concentrations compared with controls (WMD = −0.92 IU/L, 95% CI: −2.43 to 0.59, *p* = 0.234). Low‐to‐moderate heterogeneity was observed (*I* ^2^ = 39.8%) (Figure 2E). Exploratory subgroup analyses indicated a borderline significant reduction among studies with an intervention duration of ≥ 8 weeks (WMD = −1.51 IU/L, 95% CI: −3.03 to 0.00, *p* = 0.050), whereas no significant differences were observed by geographical region, intervention type, or baseline BMI (Table 4).

#### 3.4.6 Effect on Aspartate Aminotransferase

Five intervention arms from four RCTs were included in the pooled analysis of AST. Overall, apple product consumption significantly reduced serum AST concentrations (WMD = −1.56 IU/L, 95% CI: −2.69 to −0.44, *p* = 0.006), with no evidence of between‐study heterogeneity (*I* ^2^ = 0.0%) (Figure 2F). Exploratory subgroup analyses showed significant reductions in studies with an intervention duration of ≥ 8 weeks (WMD = −2.20 IU/L, 95% CI: −4.17 to −0.24, *p* = 0.028) and among overweight participants (BMI 25.0–29.9 kg/m^2^) (WMD = −2.20 IU/L, 95% CI: −4.17 to −0.24, *p* = 0.028). Although reductions were also observed in Asian and European populations and in normal‐weight participants, these subgroup effects did not reach statistical significance (Table 4).

#### 3.4.7 Effect on Alkaline Phosphatase

Three RCTs were included in the meta‐analysis of ALP. Apple product supplementation did not significantly affect serum ALP concentrations (WMD = −2.81 IU/L, 95% CI: −6.05 to 0.43, *p* = 0.089), and no between‐study heterogeneity was observed (*I* ^2^ = 0.0%) (Figure 2G). Similarly, exploratory subgroup analyses by geographical region, intervention duration, and baseline BMI did not show statistically significant effects (Table 4).

### 3.5 Publication Bias

Publication bias was evaluated using both Begg's rank correlation test and Egger's regression asymmetry test for all investigated outcomes (Figure 3). The results indicated no evidence of publication bias for any outcome, as neither Begg's nor Egger's test reached statistical significance, as provided in Table 5. Collectively, these findings suggest a low likelihood of small‐study effects or publication bias across the included meta‐analyses.

**FIGURE 3:**
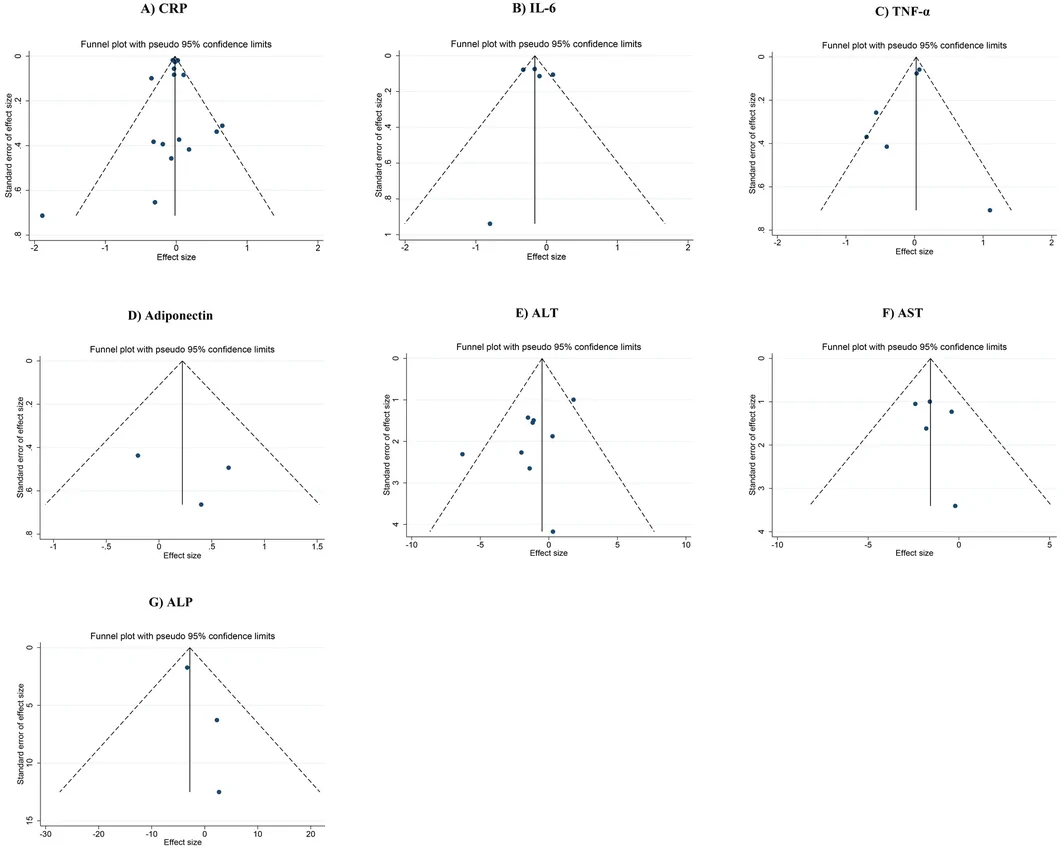
Funnel plots for the effect of apple products on: (A) CRP (mg/dL), (B) IL‐6 (pg/mL), (C) TNF‐α (pg/mL), (D) Adiponectin (mg/dL), (E) ALT (IU/L), (F) AST (IU/L), and (G) ALP (IU/L).

**TABLE 5:** Publication bias (using Begg's and Egger's tests) and sensitivity analysis.

Outcomes	Publication bias		Sensitivity analysis
	Begg's test	Egger's test	
CRP	0.392	0.730	None
IL‐6	1.000	0.925	Alzoufairi et al. 2025 (WMD: −0.21 pg/mL, 95% CI: −0.33, −0.09)
TNF‐α	1.000	0.324	None
Adiponectin	1.000	0.663	None
ALT	0.917	0.111	None
AST	0.806	0.529	Akazome et al. 2010 (A) (WMD: −1.20 IU/L, 95% CI: −2.55, 0.13)
ALP	1.000	0.251	None

Abbreviations: ALP, alkaline phosphatase; ALT, alanine aminotransferase; AST, aspartate aminotransferase; CRP, C‐reactive protein; IL‐6, interleukin‐6; TNF‐α, tumor necrosis factor‐alpha.

### 3.6 Sensitivity Analysis

Leave‐one‐out sensitivity analyses were conducted to evaluate the robustness of the pooled estimates. The overall findings for CRP, TNF‐α, adiponectin, ALT, and ALP remained stable, indicating that no individual study had a substantial influence on the corresponding pooled effect estimates. However, the pooled estimate for IL‐6 was influenced by the exclusion of the study by Alzoufairi et al. (2025), resulting in a significant reduction in IL‐6 concentrations (WMD = −0.21 pg/mL, 95% CI: −0.33 to −0.09). Similarly, for AST, omitting Akazome et al. (2010) changed the pooled estimate to a non‐significant effect (WMD = −1.20 IU/L, 95% CI: −2.55 to 0.13) (Table 5). These findings indicate that, while most outcomes were robust to the exclusion of individual studies, the pooled effects for IL‐6 and AST were moderately influenced by single studies and should therefore be interpreted with appropriate caution.

### 3.7 Linear Meta‐Regression Analysis

Linear meta‐regression showed that intervention duration was significantly associated with the pooled effect of IL‐6 (coefficient = 4.79, *p* = 0.045), suggesting that intervention duration may contribute to between‐study variation in IL‐6 effects. No significant associations between intervention duration and the pooled effects were observed for CRP, TNF‐α, adiponectin, ALT, AST, or ALP (all *p* > 0.05) (Table 6 and Figure 4).

**TABLE 6:** Linear meta‐regression and non‐linear duration‐response analysis (week).

Outcomes	Linear meta‐regression		Non‐linear duration‐response	
	Coefficient	*p*	Coefficient	*p*
CRP	−0.22	0.971	0.78	0.313
IL‐6	4.79	0.045*	−3.45	0.940
TNF‐α	−1.33	0.283	248.60	0.112
Adiponectin	8.86	0.136	20.23	NA
ALT	−0.50	0.721	6.08	0.032*
AST	−1.49	0.601	−8.04	0.202
ALP	1.56	0.372	NA	NA

*Note:* Non‐linear dose–response analyses were not applicable for adiponectin and ALP because of the limited number of available studies. Bold values with an asterisk (*) indicate statistically significant (*p* < 0.05).

Abbreviations: ALP, alkaline phosphatase; ALT, alanine aminotransferase; AST, aspartate aminotransferase; CRP, C‐reactive protein; IL‐6, interleukin‐6; NA, not available; TNF‐α, tumor necrosis factor‐alpha.

**FIGURE 4:**
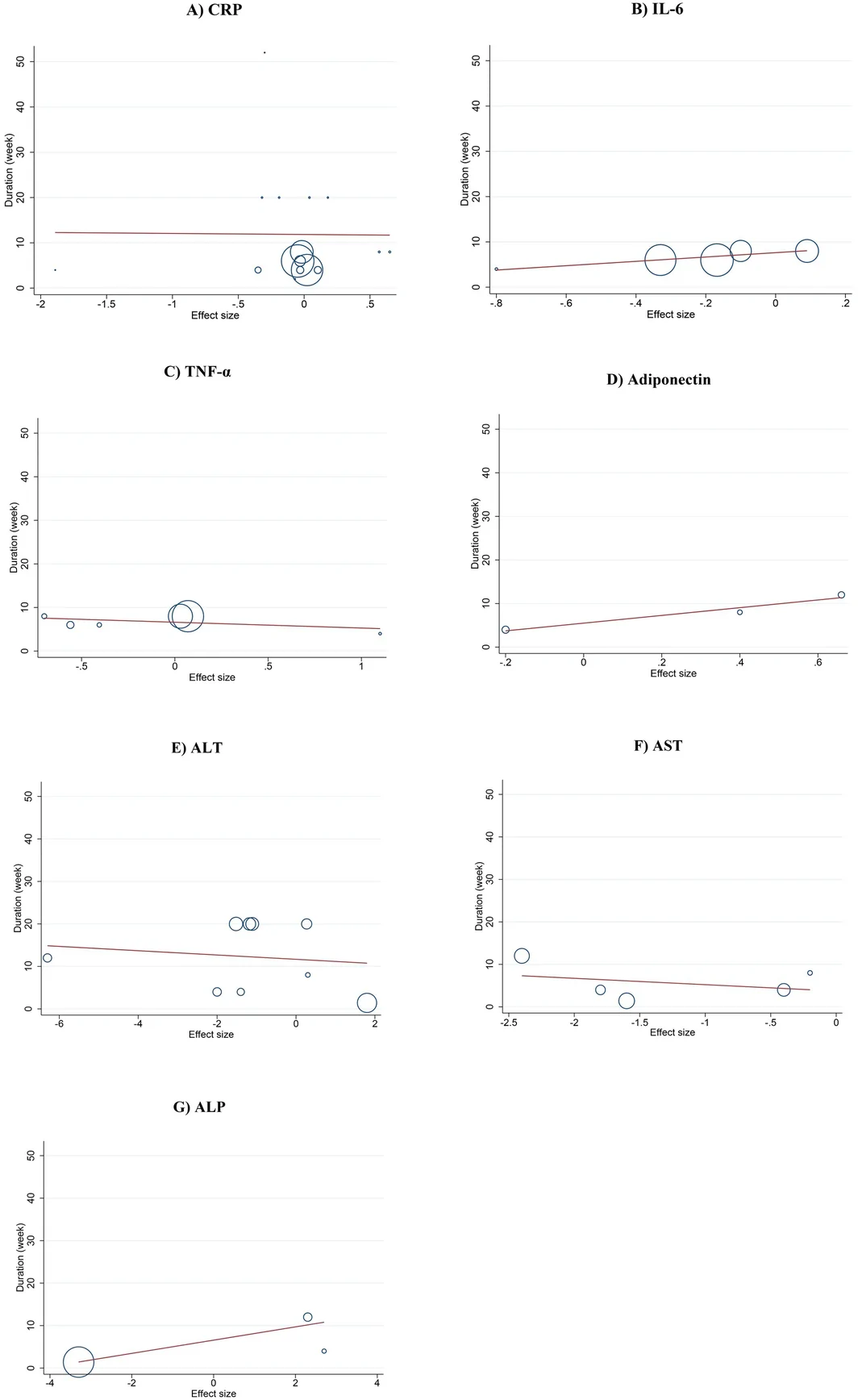
Linear dose–response relations between Apple products and absolute mean differences. Dose–response relations between duration of intervention (week) and absolute mean differences in (A) CRP (mg/dL); (B) IL‐6 (pg/mL); (C) TNF‐α (pg/mL); (D) Adiponectin (mg/dL); (E) ALT (IU/L); (F) AST (IU/L); and (G) ALP (IU/L).

### 3.8 Non‐Linear Duration–Response Analysis

The non‐linear duration‐response analysis identified a significant association between intervention duration and ALT (coefficient = 6.08, *p* = 0.032). In contrast, no significant non‐linear duration‐response associations were observed for CRP, IL‐6, TNF‐α, or AST (all *p* > 0.05). Non‐linear associations could not be estimated for adiponectin or ALP because the available data were insufficient (Figure 5 and Table 6).

**FIGURE 5:**
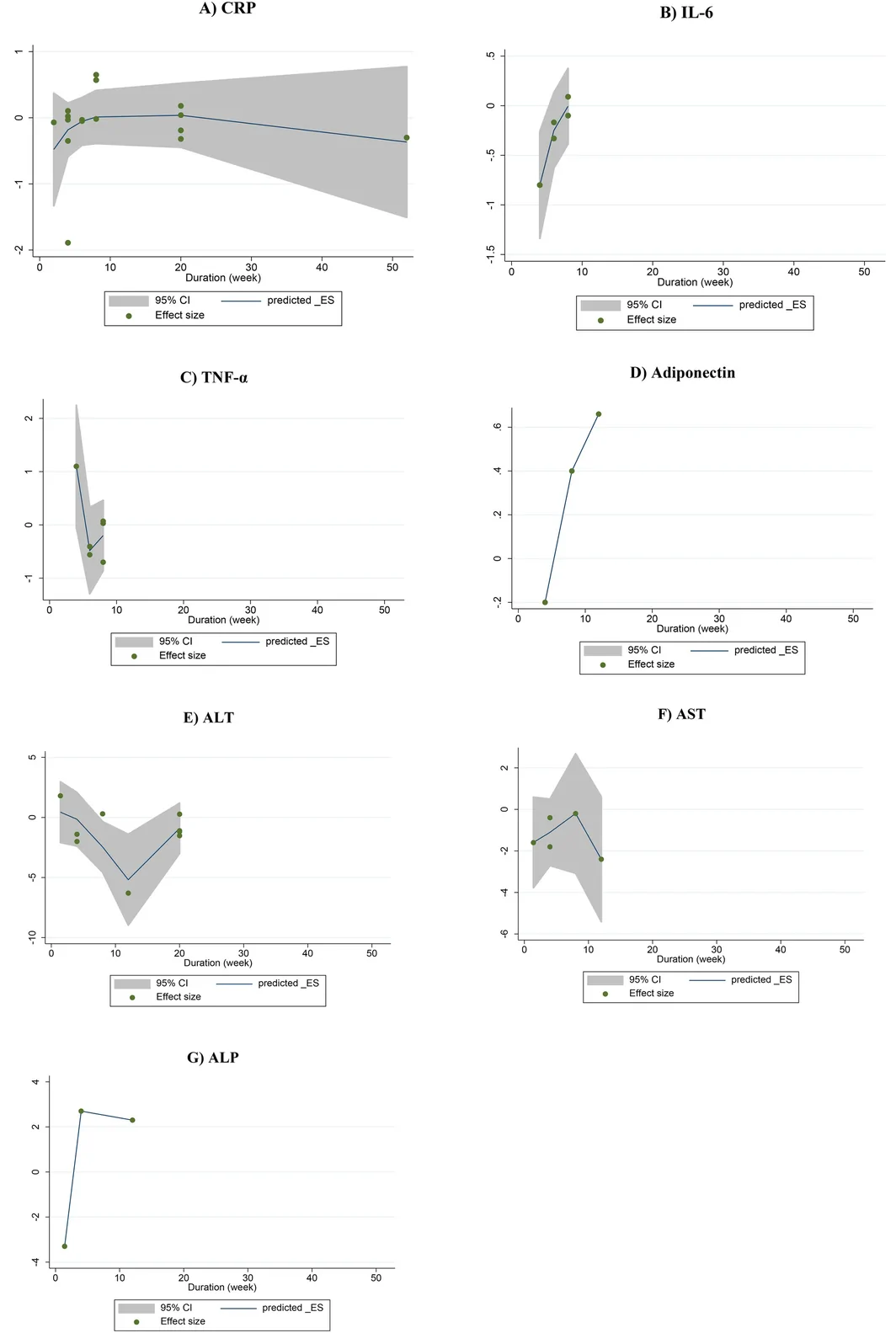
Non‐linear dose–response relations between Apple products and absolute mean differences. Dose–response relations between duration of intervention (week) and absolute mean differences in (A) CRP (mg/dL); (B) IL‐6 (pg/mL); (C) TNF‐α (pg/mL); (D) Adiponectin (mg/dL); (E) ALT (IU/L); (F) AST (IU/L); and (G) ALP (IU/L).

### 3.9 GRADE Assessment

The GRADE assessment indicated that the certainty of evidence for the effects of apple products on inflammatory biomarkers, adiponectin, and liver enzymes ranged from *Very Low* to *Moderate* (Table 7). The certainty of evidence for CRP, IL‐6, and TNF‐α was rated *Very Low*, primarily due to serious concerns about risk of bias, substantial between‐study heterogeneity, and wide confidence intervals (imprecision). The certainty of evidence for adiponectin, ALT, and ALP was rated *Low*, with downgrading mainly attributable to risk of bias and indirectness related to differences in participant characteristics. In contrast, the certainty of evidence for AST was rated *Moderate*, with the evidence downgraded only for risk of bias. Overall, the GRADE assessment indicates limited confidence in the estimated effects of apple products for most inflammatory and hepatic biomarkers.

**TABLE 7:** GRADE certainty of evidence profile of the effect of apple products on serum biomarkers of inflammation, adiponectin, and liver enzymes in adults.

Outcomes	Risk of bias	Inconsistency	Indirectness	Imprecision	Publication bias	Quality of evidence
CRP	Serious limitation^a^	Serious limitation^b^	No serious limitation	Serious limitation^c^	No serious limitation	⊕◯◯◯ Very Low
IL‐6	Serious limitation^a^	Serious limitation^b^	No serious limitation	Serious limitation^c^	No serious limitation	⊕◯◯◯ Very Low
TNF‐α	Serious limitation^a^	Serious limitation^b^	No serious limitation	Serious limitation^c^	No serious limitation	⊕◯◯◯ Very Low
Adiponectin	Serious limitation^a^	No serious limitation	No serious limitation	Serious limitation^c^	No serious limitation	⊕⊕◯◯ Low
ALT	Serious limitation^a^	No serious limitation	No serious limitation	Serious limitation^c^	No serious limitation	⊕⊕◯◯ Low
AST	Serious limitation^a^	No serious limitation	No serious limitation	No serious limitation	No serious limitation	⊕⊕⊕◯ Moderate
ALP	Serious limitation^a^	No serious limitation	No serious limitation	Serious limitation^c^	No serious limitation	⊕⊕◯◯ Low

Abbreviations: ALP, alkaline phosphatase; ALT, alanine aminotransferase; AST, aspartate aminotransferase; CRP, C‐reactive protein; IL‐6, interleukin‐6; TNF‐α, tumor necrosis factor‐alpha.

^a^ Most of the included studies have a high risk of bias.

^b^ There is a moderate level of heterogeneity (*I* ^2^ > 40%).

^c^ Participants vary in terms of clinical conditions, sex, or age group.

## 4 Discussion

### 4.1 Aim and Overall Findings

This systematic review and meta‐analysis examined the effects of a wide range of apple‐derived products and found no overall significant changes in markers such as CRP, IL‐6, TNF‐α, adiponectin, ALT, or ALP. However, several exploratory subgroup analyses suggested that the effects may vary depending on participant characteristics and intervention type. The clearest and most consistent result was a reduction in serum AST levels, an effect that was significant among trials with an intervention duration of ≥ 8 weeks, whereas the reduction observed in shorter trials did not reach statistical significance. It appeared to be more evident among Asian participants, middle‐aged or overweight individuals, and studies using apple extract preparations. Although reductions in IL‐6 and TNF‐α were observed in selected analyses, the corresponding estimates were based on a limited number of contributing trials [insert relevant study citations] and should therefore be interpreted cautiously. A modest duration‐related effect was observed for ALT, but the association did not reach strong statistical significance. In contrast, adiponectin and ALP did not appear to change meaningfully in any subgroup analysis.

The observed effects should be interpreted as responses to the respective apple‐derived interventions rather than as evidence of isolated polyphenol activity. Whole apples contain a complex food matrix comprising polyphenols, dietary fiber, organic acids, micronutrients, and other bioactive constituents that may act independently or synergistically. In addition, processing into extracts, juices, purées, or vinegar can substantially modify the composition, bioavailability, and relative contribution of these components. Therefore, differences observed across intervention types should not be interpreted simply as differences in polyphenol exposure. Alternative mechanisms may include effects related to dietary fiber, particularly pectin, gastrointestinal fermentation and gut microbial metabolism, organic acids such as acetic acid in vinegar, or interactions among multiple components of the apple matrix. Evidence from dietary‐fiber interventions suggests that fiber may influence selected inflammatory biomarkers, although effects are inconsistent across outcomes and populations; thus, such evidence provides a plausible alternative contributor rather than establishing that fiber mediates the effects observed in the present analysis.

The heterogeneity of apple‐derived interventions is also relevant to the interpretation of the pooled findings. Whole apples, extracts, juices, purées, and apple vinegar differ substantially in their composition and processing, including the relative concentrations and bioavailability of polyphenols, dietary fiber, and organic acids. Therefore, these interventions should not be interpreted as equivalent forms of polyphenol exposure. Components other than polyphenols, as well as interactions among constituents within the apple matrix, may contribute to the observed effects. Evidence from dietary‐fiber interventions provides a plausible context for such alternative mechanisms; however, it does not establish that fiber mediates the effects observed in the present analysis.

A recent systematic review by Del Bo' et al. (2026) evaluated apples and apple‐based products across a broad range of cardiometabolic and functional outcomes, including inflammatory biomarkers. The present review extends this evidence by quantitatively synthesizing data on predefined inflammatory biomarkers, adiponectin, and liver enzymes, using evidence exclusively from randomized controlled trials, while also incorporating heterogeneous interventions such as apple cider vinegar and extracts, along with an updated literature search. The overlap between the two evidence bases is therefore expected; however, the present analysis provides a more outcome‐specific quantitative synthesis of inflammatory, adipokine, and hepatic biomarkers and explicitly examines potential sources of heterogeneity across different forms of apple‐derived interventions.

### 4.2 Possible Physiological Mechanism

The biological effects of apple‐derived products on inflammatory and hepatic biomarkers observed in this meta‐analysis are primarily attributable to the rich and diverse polyphenolic composition of apple products, which includes procyanidins as the predominant fraction (approximately 45% of total polyphenols), alongside quercetin glycosides, chlorogenic acid, catechin and related flavan‐3‐ol monomers, and phloridzin (Yoshida et al. 2023), with apple vinegar additionally contributing acetic acid as a biologically active component (Chiu et al. 2020). Apple polyphenols, especially procyanidins, are known to exert anti‐inflammatory effects by suppressing key pro‐inflammatory factors, including NF‐κB and MAPK. Through this suppression, IkBa phosphorylation is inhibited, nuclear translocation of NF‐κB/p65 is prevented, and the expression and release of cytokines such as TNF‐α and IL‐6 are reduced. Consequently, levels of acute‐phase reactants, including CRP, are decrease (Han et al. 2019; Wang et al. 2020).

The greater responsiveness of obese and overweight individuals to IL‐6 and AST reductions is consistent with adiposity‐driven chronic low‐grade inflammation; hypertrophic adipose tissue triggers macrophage M1 polarization and NF‐κB‐mediated cytokine release, whose portal drainage into the liver promotes hepatic lipid peroxidation and transaminase elevation (Khanna et al. 2022; Saltiel and Olefsky 2017). These observations suggest that overweight and obese individuals may represent the population most likely to derive meaningful benefit from apple‐derived bioactive compounds. However, confirmation in trials specifically targeting these populations is warranted.

The hepatoprotective signal observed for AST in this analysis, with apple polyphenol extracts as the primary driver, is underpinned by procyanidin‐mediated promotion of acyl‐CoA oxidation enzyme activity in the liver alongside concurrent suppression of fatty acid synthase, collectively shifting hepatic metabolism away from lipogenic and steatotic pathways that damage hepatocyte integrity (Nagasako‐Akazome et al. 2007; Pour et al. 2025). This mechanism is further explained by direct clinical measurement of hepatic enzyme profiles in patients with type 2 diabetes mellitus receiving apple vinegar intervention, which yielded no significant change in AST, ALT, or ALP, consistent with the absence of meaningful visceral fat reduction in that population, suggesting that the effects of the apple‐derived intervention on transaminase levels may depend on the underlying metabolic context; however, the available evidence does not establish a specific polyphenol‐mediated mechanism (Mahmoodi et al. 2013). Annurca apple procyanidins have further demonstrated the capacity to enhance apolipoprotein A1 expression and to favor LDL receptor binding activity in hepatocytes, thereby improving intracellular lipid clearance and reducing hepatocellular stress (Tenore et al. 2018). For vinegar‐based apple products, the hepatic and metabolic mechanisms are additionally mediated by acetic acid through AMPK activation, suppression of SREBP‐1c, and inhibition of fatty acid synthase (Li et al. 2013), pathways that reduce lipogenesis, attenuate glucotoxicity‐driven hepatic inflammation, as evidenced by metabolic improvements documented in clinical trials of apple cider vinegar (Jafarirad et al. 2023; Khezri et al. 2018; Kondo et al. 2009). The consistent failure of apple products to produce a significant change in adiponectin across all included studies is consistent with the currently accepted understanding that adiponectin secretion from fat cells (adipocytes) is strongly dependent on PPARγ activation and a reduction in adipose tissue mass, suggesting that the observed effects may depend on the characteristics of the apple‐derived intervention and the metabolic context of the participants, particularly in the absence of substantial caloric restriction or weight loss (Nagasako‐Akazome et al. 2007; Astapova and Leff 2012).

### 4.3 Strengths and Limitations

This review has several methodological and analytical strengths. The exclusive inclusion of randomized controlled trials strengthens the evidentiary basis of the quantitative synthesis, while predefined outcome‐specific meta‐analyses enabled direct estimation of the effects of apple‐derived interventions on inflammatory biomarkers, adiponectin, and liver enzymes. The inclusion of different apple‐derived intervention forms allowed potential differences according to product type to be explored, and predefined subgroup and meta‐regression analyses were used to investigate potential sources of between‐study heterogeneity. Risk of bias was assessed using an established Cochrane framework, and the certainty of evidence was evaluated using GRADE, supporting transparent interpretation of the pooled findings.

Several limitations should be considered when interpreting the findings of this meta‐analysis. Although only RCTs were included, many studies were judged to have a high risk of bias, particularly in the domains of randomization and deviations from intended interventions, which may reduce confidence in some pooled estimates. In addition, considerable clinical and methodological heterogeneity existed across studies regarding participant characteristics, intervention duration, dosage, and the type of apple‐derived product evaluated, including whole apples, juices, polyphenol extracts, pomace, purees, and apple cider vinegar, each differing in bioactive composition and bioavailability. Several outcomes, particularly adiponectin, AST, and ALP, were assessed in a limited number of trials, restricting statistical power and the reliability of subgroup and sensitivity analyses. Furthermore, most interventions were relatively short in duration, limiting conclusions about the long‐term effects of apple‐derived products on inflammatory and hepatic biomarkers. In addition, an additional limitation is the heterogeneity of the apple‐derived interventions. Whole apples, extracts, juices, purées, and apple vinegar differ in composition, processing, dose, and bioavailability of their constituent compounds. Consequently, pooled estimates should be interpreted as effects of the respective apple‐derived interventions rather than as evidence for the effect of a single bioactive component. Finally, potentially important modifiers of treatment response, such as habitual dietary intake, physical activity, medication use, gut microbiota composition, and inter‐individual differences in polyphenol metabolism, were inconsistently reported and could not be adequately evaluated. Therefore, the present findings should be interpreted with appropriate caution and confirmed by larger, high‐quality, long‐term RCTs using standardized apple‐derived interventions.

### 4.4 Future Research Direction

Future research should prioritize adequately powered, well‐designed RCTs using standardized apple products with quantified polyphenol and other bioactive compound content, longer intervention durations, and clearly defined doses to establish dose–response relationships and long‐term efficacy. Direct comparisons of whole apples, extracts, juices, pomace, and apple cider vinegar may help clarify the relative contributions of polyphenols, fiber, and acetic acid. Studies should also investigate sources of inter‐individual variability by considering metabolic phenotype, geographical region, and gut microbiota composition, particularly among populations with obesity, metabolic dysfunction, non‐alcoholic fatty liver disease, or elevated inflammatory biomarkers. Finally, mechanistic studies assessing pathways such as NF‐κB and Nrf2, together with gut microbiota–mediated polyphenol metabolism, may help explain heterogeneous clinical responses and strengthen the translation of mechanistic findings into clinical applications.

## 5 Conclusion

Apple and apple‐derived products did not significantly improve most evaluated inflammatory biomarkers, adiponectin, or liver enzymes in adults, although a significant reduction in AST was observed. Exploratory subgroup analyses suggested that effects may vary by apple product type, intervention duration, and participant characteristics. Given the low to very low certainty of evidence for most outcomes, these findings should be interpreted cautiously. Further well‐designed, adequately powered RCTs with standardized apple interventions and longer follow‐up are warranted to clarify their potential effects on inflammation, adipokine regulation, and hepatic biomarkers.

## Author Contributions

**Sahel Hamednia:** methodology, software, data curation, investigation, validation, funding acquisition, visualization, resources. **Mehdi Karimi:** conceptualization, methodology, software, investigation, validation, supervision, funding acquisition, visualization, project administration, resources, writing – original draft, writing – review and editing. **Camellia Akhgarjand:** conceptualization, methodology, software, investigation, validation, funding acquisition, visualization, project administration, resources, writing – original draft. **Elaheh Dehghani:** methodology, software, data curation, investigation, validation, funding acquisition, visualization, resources. **Melina Todehkharman:** writing – review and editing, writing – original draft, funding acquisition, conceptualization, software, resources, validation, investigation. **Mohammad Mehdi Khaleghi:** methodology, software, data curation, investigation, validation, funding acquisition, visualization, resources. **Omid Asbaghi:** conceptualization, methodology, software, data curation, investigation, validation, formal analysis, supervision, funding acquisition, visualization, project administration, resources, writing – review and editing. **Ali Hosseini:** supervision, project administration, methodology, investigation, validation. **Moslem Naderian:** methodology, validation, supervision, funding acquisition, project administration.

## Funding

This work did not receive any specific grant from funding agencies in the public, commercial, or not‐for‐profit sectors.

## Ethics Statement

The authors have nothing to report.

## Consent

The authors have nothing to report.

## Conflicts of Interest

The authors declare no conflicts of interest.

## Supporting information


**Supplementary Table 1.** Search keywords and search strategy.
**Supplementary Table 2.** Query boxes of each database based on the Title & Abstract (search date: August 1, 2026).

## Data Availability

All data used in this meta‐analysis were extracted from published studies. The datasets supporting the findings of this study are available from the original sources, which are cited in the manuscript. Additional information can be provided by the corresponding author upon reasonable request.
