# Supplementary material for: Effects of Apple and Apple‐Derived Products on Serum Inflammatory Biomarkers, Adiponectin, and Liver Enzymes in Adults: A Systematic Review and Meta‐Analysis of Randomized Controlled Trials
Source: Food Sci Nutr. 2026 Nov 11;14(11):e72547. doi: 10.1002/fsn3.72547 (PMC13755682; doi:10.1002/fsn3.72547)
Supplement: Supplementary file 1 [file FSN3-14-e72547-s001.docx]

**Supplementary Materials**

**Title:**

**Effects of Apple and Apple-Derived Products on Serum Inflammatory Biomarkers, Adiponectin, and Liver Enzymes in Adults: A Systematic Review and Meta-Analysis of Randomized Controlled Trials**

**Supplementary Table 1.** Search keywords and search strategy

|  | **Search Keywords** |
| --- | --- |
| **Intervention** | “apple*” OR Malus OR “Malus domestica” |
| **Outcomes** | “inflamm*” OR Inflammation OR “C-reactive protein” OR CRP OR “Interleukin-6” OR IL-6 OR “Tumor Necrosis Factor-alpha” OR TNF-alpha OR TNF-α OR adiponectin OR adipokine* OR “Alanine aminotransferase” OR “Alanine Transaminase” OR ALT OR “Aspartate Aminotransferase” OR “Aspartate transaminase” OR AST OR “Alkaline Phosphatase” OR “Alkaline phosphomonoesterase” OR ALP OR “liver enzyme*” OR “hepatic biomarker*” OR “liver function” |
| **Study designs** | “Controlled trial” OR “clinical trial” OR trial OR RCT OR Randomized OR random* OR placebo OR intervention |

**Supplementary Table 2.** Query boxes of each database based on the Title & Abstract. (search date: 01-August-2026)

| **Databases** | **Search** | **Search line** | **No.** |
| --- | --- | --- | --- |
| **PubMed** | Title/Abstract | (("apple*"[Title/Abstract] OR Malus[Title/Abstract] OR "Malus domestica"[Title/Abstract]) AND ("inflamm*"[Title/Abstract] OR Inflammation[Title/Abstract] OR "C-reactive protein"[Title/Abstract] OR CRP[Title/Abstract] OR "Interleukin-6"[Title/Abstract] OR IL-6[Title/Abstract] OR "Tumor Necrosis Factor-alpha"[Title/Abstract] OR TNF-alpha[Title/Abstract] OR TNF-α[Title/Abstract] OR adiponectin[Title/Abstract] OR adipokine*[Title/Abstract] OR "Alanine aminotransferase"[Title/Abstract] OR "Alanine Transaminase"[Title/Abstract] OR ALT[Title/Abstract] OR "Aspartate Aminotransferase"[Title/Abstract] OR "Aspartate transaminase"[Title/Abstract] OR AST[Title/Abstract] OR "Alkaline Phosphatase"[Title/Abstract] OR "Alkaline phosphomonoesterase"[Title/Abstract] OR ALP[Title/Abstract] OR "liver enzyme*"[Title/Abstract] OR "hepatic biomarker*"[Title/Abstract] OR "liver function"[Title/Abstract])) AND ("Controlled trial"[Title/Abstract] OR "clinical trial"[Title/Abstract] OR trial[Title/Abstract] OR RCT[Title/Abstract] OR Randomized[Title/Abstract] OR random*[Title/Abstract] OR placebo[Title/Abstract] OR intervention[Title/Abstract]) | 125 |
| **WoS** | Topic (Searches title, abstract, keyword plus, and author keywords) | ( TITLE-ABS-KEY (“apple*” OR Malus OR “Malus domestica”) AND TITLE-ABS-KEY (“inflamm*” OR Inflammation OR “C-reactive protein” OR CRP OR “Interleukin-6” OR IL-6 OR “Tumor Necrosis Factor-alpha” OR TNF-alpha OR TNF-α OR adiponectin OR adipokine* OR “Alanine aminotransferase” OR “Alanine Transaminase" OR ALT OR “Aspartate Aminotransferase" OR “Aspartate transaminase” OR AST OR “Alkaline Phosphatase” OR “Alkaline phosphomonoesterase” OR ALP OR “liver enzyme*” OR “hepatic biomarker*” OR “liver function”) AND TITLE-ABS-KEY ( "controlled trial" OR "clinical trial" OR trial OR rct OR randomized OR random* OR placebo OR intervention ) ) | 257 |
| **Scopus** | TITLE-ABS-KEY | ( TITLE-ABS-KEY (“apple*” OR Malus OR “Malus domestica”)  AND  TITLE-ABS-KEY (“inflamm*” OR Inflammation OR “C-reactive protein” OR CRP OR “Interleukin-6” OR IL-6 OR “Tumor Necrosis Factor-alpha” OR TNF-alpha OR TNF-α OR adiponectin OR adipokine* OR “Alanine aminotransferase” OR “Alanine Transaminase" OR ALT OR “Aspartate Aminotransferase" OR “Aspartate transaminase” OR AST OR “Alkaline Phosphatase” OR “Alkaline phosphomonoesterase” OR ALP OR “liver enzyme*” OR “hepatic biomarker*” OR “liver function”) AND TITLE-ABS-KEY (“Controlled trial" OR "clinical trial" OR trial OR RCT OR Randomized OR random* OR placebo OR intervention) | 388 |
| ***All*** |  | Duplicates = 191 | 770 |
